# Machine learning identifies the role of SMAD6 in the prognosis and drug susceptibility in bladder cancer

**DOI:** 10.1007/s00432-024-05798-z

**Published:** 2024-05-20

**Authors:** Ziang Chen, Yuxi Ou, Fangdie Ye, Weijian Li, Haowen Jiang, Shenghua Liu

**Affiliations:** 1 Department of Urology, Huashan Hospital, Fudan University, Shanghai, China; 2 Fudan Institute of Urology, Huashan Hospital, Fudan University, Shanghai, China; 3 National Clinical Research Center for Aging and Medicine, Huashan Hospital, Fudan University, Shanghai, China

**Keywords:** Bladder cancer, Machine learning, Tumor microenvironment, Drug sensitivity, Therapeutic target, Immunotherapy

## Abstract

**Background:**

Bladder cancer (BCa) is among the most prevalent malignant tumors affecting the urinary system. Due to its highly recurrent nature, standard treatments such as surgery often fail to significantly improve patient prognosis. Our research aims to predict prognosis and identify precise therapeutic targets for novel treatment interventions.

**Methods:**

We collected and screened genes related to the TGF-β signaling pathway and performed unsupervised clustering analysis on TCGA-BLCA samples based on these genes. Our analysis revealed two novel subtypes of bladder cancer with completely different biological characteristics, including immune microenvironment, drug sensitivity, and more. Using machine learning classifiers, we identified SMAD6 as a hub gene contributing to these differences and further investigated the role of SMAD6 in bladder cancer in the single-cell transcriptome data. Additionally, we analyzed the relationship between SMAD6 and immune checkpoint genes. Finally, we performed a series of in vitro assays to verify the function of SMAD6 in bladder cancer cell lines.

**Results:**

We have revealed two novel subtypes of bladder cancer, among which C1 exhibits a worse prognosis, lower drug sensitivity, a more complex tumor microenvironment, and a ‘colder’ immune microenvironment compared to C2. We identified SMAD6 as a key gene responsible for the differences and further explored its impact on the molecular characteristics of bladder cancer. Through in vitro experiments, we found that SMAD6 promoted the prognosis of BCa patients by inhibiting the proliferation and migration of BCa cells.

**Conclusion:**

Our study reveals two novel subtypes of BCa and identifies SMAD6 as a highly promising therapeutic target.

## Introduction

BCa ranks as the second most common malignant tumor in the urinary system. As of 2020, it annually accounts for approximately 570,000 new cases and 210,000 deaths worldwide (Sung et al. 2021). With the rise in tobacco consumption and smoking rates, the incidence of BCa is also increasing (Lenis et al. 2020). Classified by stage, BCa can be categorized into non-muscle-invasive bladder cancer (NMIBC) and muscle-invasive bladder cancer (MIBC) (Witjes et al. 2021). Among patients diagnosed with non-muscle-invasive bladder cancer, one-third to three-quarters experience recurrence within five years after surgery, and about 15% progress to MIBC (Bajorin et al. 2021). Surgery and chemotherapy are traditional means of managing BCa. However, due to its recurrent and progressive nature, the prognosis and survival rates are generally poor (Balar et al. 2021). With the emergence of targeted therapy and immunotherapy, identifying reliable treatment targets is essential for a comprehensive improvement in BCa management (de Jong et al. 2021). On the other hand, a deep exploration of the biological mechanisms underlying the occurrence and development of BCa is crucial for developing new treatment approaches and extending the survival time of advanced-stage patients.

The TGF-β signaling pathway is a complex and highly conserved signaling pathway within living organisms (Derynck et al. 2021). It has been demonstrated to be associated with several hallmarks of cancer, such as evasion of immune surveillance, resistance to cell death, and activation of invasion and metastasis (Massagué, 2008). The TGF-β signaling pathway has a broad impact, with increased TGF-β expression observed in almost all solid tumor cells. The TGF-β latent complex binds to TGF-β receptors on the cell membrane, leading to phosphorylation and activation of SMAD2 and SMAD3 (Derynck and Budi 2019). Subsequently, these SMADs are transported into the cell nucleus, where they bind to transcription factors, activating or inhibiting the transcription of target genes (Syed 2016). Additionally, the TGF-β signaling pathway is closely related to pathways associated with cancer progression, such as Myc, Notch, Wnt, and PI3K/AKT, among others (Bierie and Moses 2006). In urine samples collected by some researchers, samples from BCa show higher expression of TGFβ-mRNA, TGFβ1 protein, and its receptors compared to samples from the healthy group (Lu et al. 2022). Epithelial-mesenchymal transition (EMT) refers to the process in which epithelial cells lose their original biological characteristics and acquire features of mesenchymal cells (Goulet et al. 2019). EMT promotes the invasive and migratory activity of cancer cells. Studies have found that TGF-β can promote EMT in BCa by activating transcription factors such as ZEB1/2 through the SMAD3/4 pathway, activating stromal genes, and inhibiting epithelial genes (Hao et al. 2019). Researchers have also found that the TGF-β signaling pathway is significantly upregulated in MIBC compared to NMIBC, further indicating its association with BCa progression (Katoh and Nakagama 2014). The TGF-β signaling pathway can also influence tumor progression and treatment by regulating the tumor microenvironment (Najafi et al. 2019). TGF-β has been demonstrated to exert an immunosuppressive role within the microenvironment, inhibiting the production of the cytokine IFNγ, which promotes the proliferation of CD8 + T cells (Yang et al. 2010). Furthermore, TGF-β can inhibit dendritic cell immune surveillance and antigen presentation (Tauriello et al. 2022). These characteristics suggest that the TGF-β signaling pathway may impact immune checkpoint inhibitor (ICI) therapy.

This study utilized consensus clustering in the TCGA-BLCA cohort to identify two TGF-β related subtypes with distinct clinical and molecular characteristics. Subsequently, machine learning classifiers were employed to identify the gene SMAD6 as a causative factor for these differences. Integrating single-cell and pan-cancer transcriptome data, we analyzed the potential role of SMAD6 in various cancers, including BCa, revealing that lower expression of SMAD6 in BCa is associated with a worse prognosis. Finally, these findings were validated in in vitro experiments. Our research offers novel insights into the influence of the TGF-β signaling pathway on BCa and identifies SMAD6 as a potential therapeutic biomarker.

## Materials and methods

### Data collection and preprocessing

Various BCa datasets were involved in this study, TCGA-BLCA (n = 406) was achieved from UCSC Xena (https://xenabrowser.net/). GSE32894 and corresponding clinical data were retrieved from the Gene Expression Omnibus (GEO) (https://www.ncbi.nlm.nih.gov/geo/). All raw mRNA sequencing data were filtered and normalized before analysis. The single-cell RNA sequencing data was downloaded from SRP280327 in the Sequence Read Archive (https://www.ncbi.nlm.nih.gov/sra), which contained 8 bladder carcinoma tumor samples and 3 normal tissues (Chen et al. 2020b). The raw FASTQ data underwent filtering and read alignment using CellRanger (v.3.0.1). The single-cell data underwent rigorous quality control before analysis.

### Identification of SMAD6 as a prognostic gene

27 TGF-β signaling pathway genes were acquired from the MSigDB database (https://www.gsea-msigdb.org/gsea/index.jsp). To further examine the various expression features of TGF-β signaling pathway genes in BCa, we performed consensus clustering using the k-means method with the ConsensusClusterPlus R package (version 1.64.0) to classify individuals with BCa. Kaplan–Meier (KM) survival curves and optimal cutoff values were generated using the survival (version 3.5.5) and survminer R package (version 0.4.9) to compare outcomes between the two clusters. Principal component analysis (PCA) was employed to validate the clustering tendency. The expression differences of genes related to the TGF-β signaling pathway form the basis for the biological phenotype differences between clusters. Machine learning classifiers can determine which genes bear the primary responsibility for these differences based on expression matrices. The LASSO-LR algorithm, random-forest algorithm, Boruta algorithm, XGboost algorithm, and svmREF (Li et al. 2023b) algorithm were utilized to screen feature variables, specifically SMAD6.

### TME immunological characteristics analysis

The immune score, stromal score, and tumor purity of malignant tumors were quantified based on the ESTIMATE R package (version 1.0.13). We integrated four algorithms to compute the abundance of immune infiltrating cells based on the IOBR R package (version 0.99.9) (Zeng et al. 2021). Besides, the single-sample gene set enrichment analysis (ssGSEA) was implemented to calculate enrichment scores based on the gene set variation analysis (GSVA) package (version 1.48.3) (Hänzelmann et al. 2013). We performed the correlation analysis to explore the relationship between SMAD6 and immune checkpoint genes, as well as immune cell infiltration. The package GseaVis (version 0.0.9) was used to visualize the outcomes of the enrichment analysis.

### Treatment-related analysis

The tumor immune dysfunction and exclusion (TIDE) was employed to infer the responses to ICI therapy in BCa. All pharmacogenomic data were obtained from the Genomics of Drug Sensitivity in Cancer (GDSC, https://www.cancerrxgene.org/), and eight chemotherapeutic drugs were selected for subsequent analysis. The drug sensitivity reckoned by the R package oncoPredict (version 0.2) was utilized to predict the drug responses (Maeser et al. 2021).

### Single-cell transcriptomics sequencing data analysis

We created Seurat objects for total cell types belonging to the single-cell data expression matrix based on the R package Seurat (version 4.3.0.1), and the top 2000 genes were selected as the most variable features for normalizing the single-cell RNA data. Next, we used ScaleData and RunPCA functions to get the number of principal components (PC), UMAP (unified manifold approximation and projection) dimensionality reduction was used to summarize the top principal components. Finally, every cell cluster was annotated based on classical marker genes for known cell types. The Scissor R package (version 2.1.0) was employed to identify cells that are associated with prognosis (Sun et al. 2022).

### Cell culture, transfection, and interference

The study utilized human bladder cancer cells (UMUC3 and T24 cells) and human bladder cells (SV-HUC-1) obtained from the Cell Bank of the Chinese Academy of Sciences (Shanghai, China). We cultured the cells in high glucose DMEM with 10% fetal bovine serum (Gibco, USA) and 1% streptomycin/penicillin (Thermo Fisher Scientific, USA) in an incubator at 37℃ and 5% CO2. The cells were transferred upon reaching a cell density of 70–80%, and the medium was changed daily.

The shRNA targeting SMAD6 (shSMAD6) lentivirus was purchased from GeneChem (shanghai, China) to suppress the SMAD6 gene of T24 and UMUC3 cells. The shSMAD6 was transfected into T24 and UMUC3 cells with polyethylene, then the cells were screened with puromycin.

### Quantitative real‑time polymerase chain reaction (qRT‑PCR)

RNA was extracted from the cell lines using TRIzol reagent (Invitrogen, Carlsbad, CA). Subsequently, cDNA was synthesized using the reverse transcription kit supplied by QIAGEN (Japan). To assess the mRNA expression levels of SMAD6, a cDNA mixture was loaded onto a 96-well microplate and amplified following standardized protocols. The relative expression of mRNAs was determined using the 2^−ΔΔCt^ method with GAPDH as the internal reference. Primer information is shown in Supplementary Table 1.

### Colony formation assay

UMUC3 and T24 cells were seeded onto 12-well plates at a density of 100 cells per well and allowed to culture for 2 weeks until the formation of cell colonies. Subsequently, the cells were washed three times with phosphate-buffered solution (PBS) (Yeasen, China), fixed with 4% methanol for 15 min, stained with 0.5% crystal violet solution for 30 min, and analyzed using ImageJ software.

### Wound healing assay

To analyze cell migration, a wound healing assay was conducted. UMUC3 and T24 cells were inoculated in a 6-well plate and cultured until reaching 70%-80% density. The cell monolayer was gently scratched using the tip of a sterile 200µL pipette after removing the medium. Subsequently, the wells were rinsed twice with PBS, and serum-free medium was added for continued culture. Images were captured at 0-, 12-, and 24-h post-scratching and analyzed using ImageJ software.

### Cell viability detection

A CCK-8 assay kit (Biosharp, China) was used to assess cell viability. After the intervention, cells were seeded on 96-well plates and incubated at 37℃ with 5% CO2. UMUC3 and T24 cells were treated with a diluted CCK-8 solution for 2 h. The absorbance values at 450 nm were quantified using a microplate reader. (Thermo Fisher Scientific, USA).

### Transwell migration and invasion assay

The transwell assays were performed to observe the migration and invasion ability. T24 and UMUC3 cells were seeded at a density of 2 X 10^4^ cells per well in the upper chambers, with 200µL serum-free medium, while the lower chambers contained 800µL of medium supplemented with 10% serum. To perform the invasion assay, 50 mg/L Matrigel glue was covered in the upper chambers. After placing the transwell chambers (Corning, USA) in a 37℃, 5% CO2 incubator for 48 h, 4% methanol was used to fix cells for 30 min, and 0.5% crystal violet was employed to stain for 30 min. Finally, the results can be obtained by taking photographs and counting.

### Statistical analysis

Wilcoxon test was employed to assess the difference between various groups, as well as p-value calculations. The log-rank test and Kaplan–Meier curve were used to perform survival analysis, and we excluded samples with a survival period of less than 30 days to ensure accuracy. The statistical test used for correlation analysis is the Pearson correlation test. The experiment data were presented as the mean ± SD. GraphPad Prism 8 software was employed to analyze these experiment data; the difference was considered statistically significant when the p-value was less than 0.05. The significance of the differences was presented as follows: * p < 0.05; **p < 0.01; ***p < 0.001; ****p < 0.0001.

## Results

### Two distinct clusters of the TGF-β signaling pathway

**Fig. 1:**
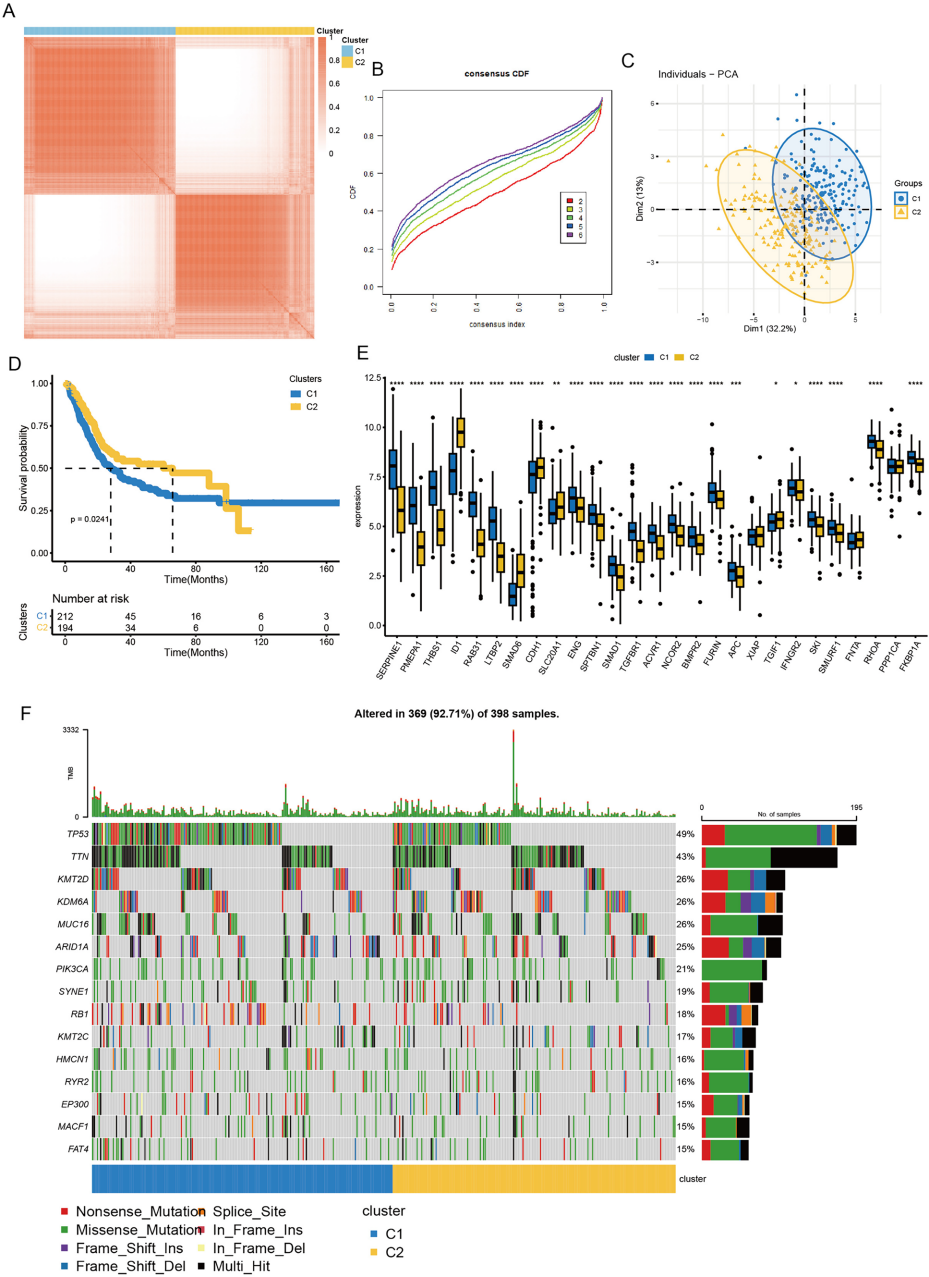
Consensus clustering analysis of TGF-β signaling pathway in BCa. **A** consensus clustering of BCa subtypes: cluster1 and cluster2. **B** the optimal number of clusters. **C** Visualization of TGF-β clusters by PCA. **D** Kaplan–Meier curves (OS) comparing two clusters in BCa. **E** The expression of TGF-β related genes between two clusters. **F** Demonstration of the top genes with the highest mutation frequency in two clusters. Wilcoxon test: ^*^P < 0.05, ^**^P < 0.01, ^***^P < 0.001, ^****^P < 0.0001

We evaluated the clustering proficiency of the TGF-β signaling pathway and depicted it (Fig. 1A), the ConsensusClusterPlus package was employed to determine the optimal cluster number and appraise the stability of clustering. Our analysis revealed that k = 2 was the optimal choice (Fig. 1B). Subsequently, the clustering tendency was assessed via PCA. C1 was denoted by the pink circle, while C2 was represented by the cyan circle. Noticeable separation of TGF-β signaling pathway clusters was observed, indicating a robust consensus cluster outcome (Fig. 1C).

We further investigated the overall survival of bladder cancer patients in C1 and C2. The Kaplan–Meier curve illustrated that C2 exhibited higher and prolonged survival compared to those in C1 (Fig. 1D, p < 0.0241). Additionally, there are significant differences in TGF-β signaling pathway genes expressed between the two clusters (Fig. 1E). The heatmap of mutational distribution in C1 and C2 was shown (Fig. 1F). As a biomarker of tumorigenesis (Olivier et al. 2010), TP53 mutation frequency in C1 was higher than in C2. In conclusion, the TGF-β signaling pathway has a significant correlation with the prognosis of bladder cancers.

### Biology and immune features of two subtypes

**Fig. 2:**
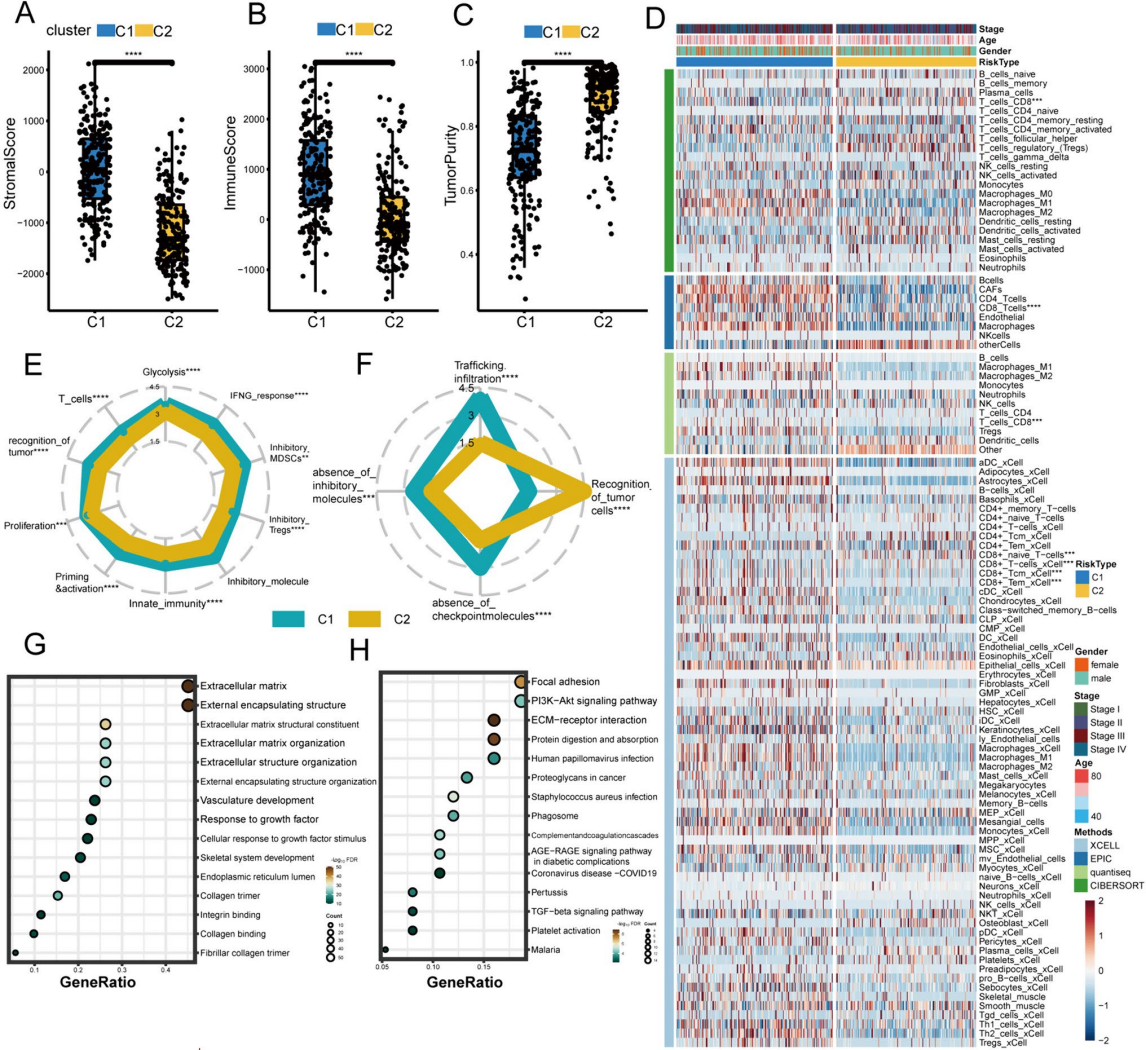
Immune landscape of the clusters and enrichment analysis. **A**–**C** The difference of stromalscore (**A**), immunesore (**B**), tumorpurity (**C**) between two clusters. **D** The landscape of immune infiltration of BCa subtypes. **E**–**F** The distribution of immune processes (**E**) and immune evasion (**F**) signatures among two clusters. **G**–**H** The GO (**G**) and KEGG (**H**) enrichment analysis of those genes upregulated in C1. (*GO* Gene Ontology; *KEGG* Kyoto Encyclopedia of Genes) Wilcoxon test: ^*^P < 0.05, ^**^P < 0.01, ^***^P < 0.001, ^****^P < 0.0001

To investigate the immune characteristics of C1 and C2, we calculated the StromalScore, ImmuneScore, and TumorPurity of the two clusters (Fig. 2A–C). Among these results, the TME scores of C1 were higher than those of C2, but the tumor purity of C2 was higher than that of C1. Then, we employed the IOBR package to compare the immune infiltration of the two clusters based on 4 algorithms (Fig. 2D). These samples in C1 had more TME abundance, especially macrophages, and CAFs (carcinoma-associated fibroblasts). Interestingly, in the calculations of most algorithms (except CIBERSORT), the infiltration of cytotoxic T cells in C1 is more abundant than in C2. Moreover, ssGSEA deduced that the immune components of C1 were predominantly inhibitory, and its immune microenvironment was ‘colder’ compared to C2 (Fig. 2E–F) (van Dijk et al. 2019). This predicted different responses between the C1 and C2 subtypes when facing ICI treatment.

GO (Gene Ontology) and KEGG (Kyoto Encyclopedia of Genes) analysis were performed to investigate the functions of those genes overexpressed in C1. The GO analysis yielded that these genes were enriched in biological processes related to the extracellular matrix, such as extracellular matrix formation and vasculature development. (Fig. 2G). The KEGG results indicated that these genes were enriched in signaling pathways such as PI3K-Akt and TGF-β signaling pathways (Fig. 2H).

### GSEA analysis and metabolic characteristics of BCa subtypes

**Fig. 3:**
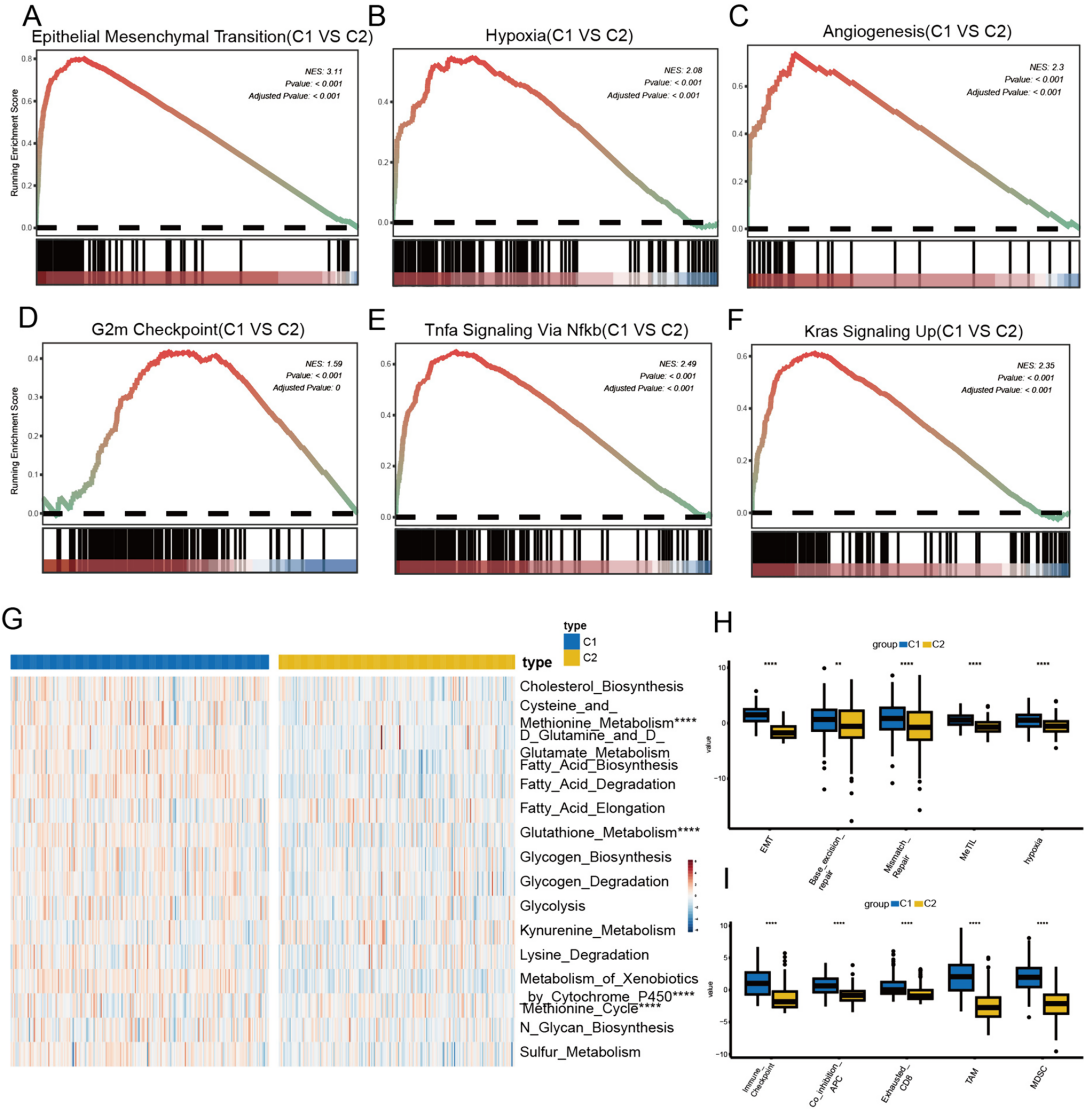
The biological differences between the two clusters include metabolism and immunity. **A**–**F** GSEA analysis investigates the biological functions of C1. **G** The metabolism landscape of the various subtypes of BCa. **H**–**I** The distribution of molecular characteristics (**H**) and immune microenvironment signatures (**I**) among two clusters. (*GSEA* Gene Set Enrichment Analysis) Wilcoxon test: ^*^P < 0.05, ^**^P < 0.01, ^***^P < 0.001, ^****^P < 0.0001

Differential analysis was performed to identify genes exhibiting differential expression between the two subtypes, GSEA analysis revealed that biological processes significantly enriched in C1, including EMT, hypoxia, and angiogenesis (Fig. 3A–C, p < 0.001). These biological characteristics were widely demonstrated to be closely associated with tumor progression and metastasis. Besides, GSEA analysis yielded that TNFα-NFκB and Kras signaling pathways were upregulated in C1 (Fig. 3D–F, p < 0.001). Then the metabolism characteristics of the two subtypes were unraveled. The heatmap showed that C1 exhibited more vigorous methionine and glutathione metabolism compared to C2 (Fig. 3G, p < 0.0001). Some studies indicated that methionine metabolism was associated with platinum drug resistance in bladder cancer (Singh et al. 1995), while glutathione metabolism was associated with the level of hypoxia in the tumor microenvironment (Tuleta et al. 2014). The analysis results of the IOBR package also underscored that compared to C2, C1 has a lower immune microenvironment temperature (Fig. 3H–I).

### Clinical correlations analysis of TGF-β signaling clusters

**Fig. 4:**
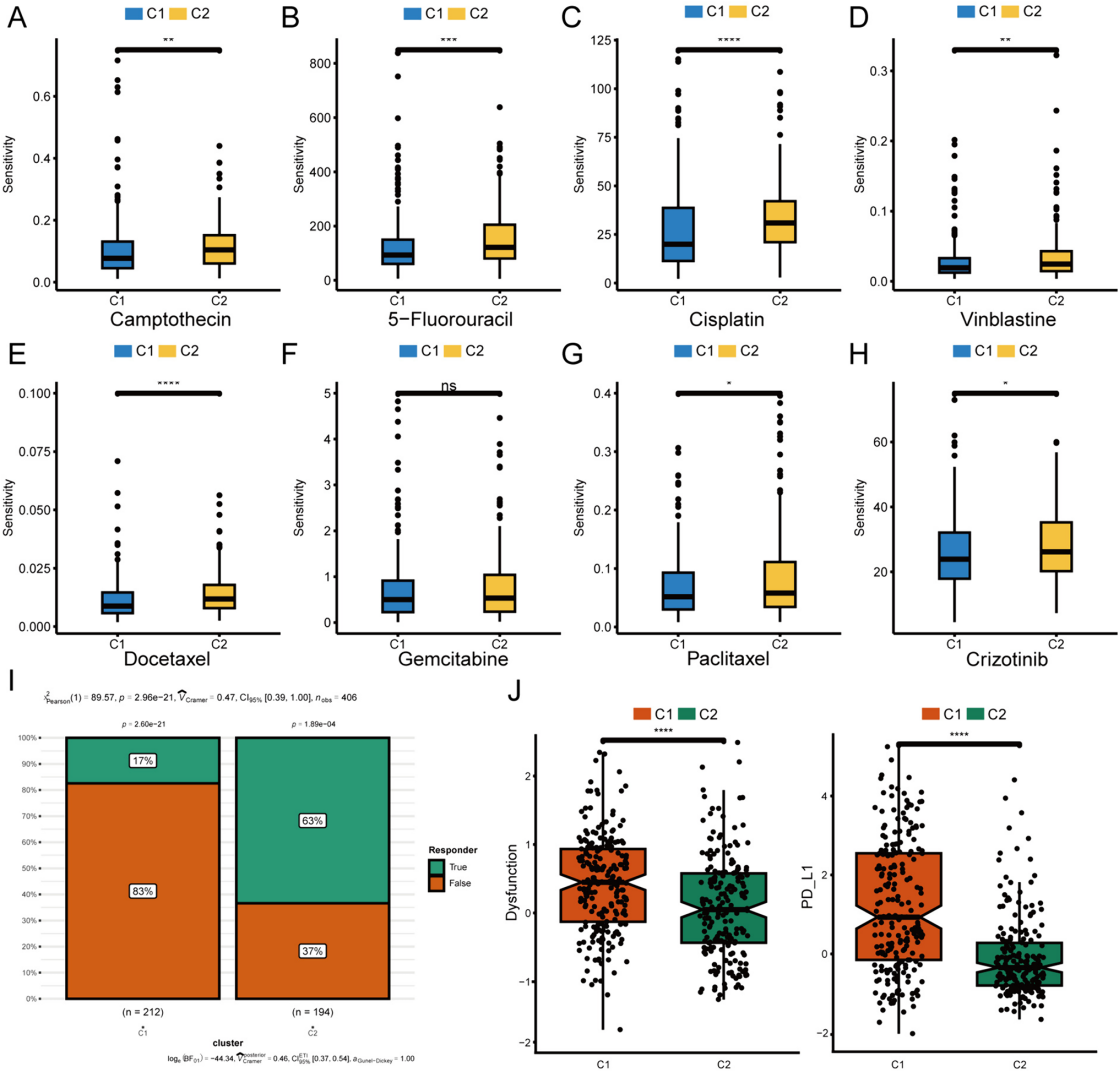
The differences in sensitivity to chemotherapy and immunotherapy between the two clusters. **A–H** The sensitivity comparison of eight commonly applied chemotherapy drugs. **I** Prediction of the response of BCa subtypes to ICI therapy. **J** The difference of ICI therapy-related signatures among BCa subtypes. (*TIDE* Tumor Immune Dysfunction and Exclusion) Wilcoxon test: ^*^P < 0.05, ^**^P < 0.01, ^***^P < 0.001, ^****^P < 0.0001

To further validate the clinical significance of the TGF-β signaling pathway, we uncovered the clinical characteristic differences between the two clusters. The results indicated that C1 exhibited lower susceptibility to many commonly used chemotherapy drugs (Fig. 4A–H). Cisplatin, being the most commonly applied chemotherapy drug for bladder cancer, exhibited significantly lower sensitivity in C1 compared to C2. Besides, 5-fluorouracil, docetaxel, and paclitaxel were also less sensitive. To test the role of TGF-β signaling in immunotherapy, we employed the TIDE algorithm to estimate the immunotherapy responses of two clusters. TIDE yielded that a larger proportion of patients in C1 were resistant to immunotherapy (Fig. 4I), and C1 had higher immune escape potential (Fig. 4J). These outcomes suggested that the TGF-β signaling pathway was associated with therapeutic effects in bladder cancer patients.

### Identification of SMAD6 as a prognostic gene

**Fig. 5:**
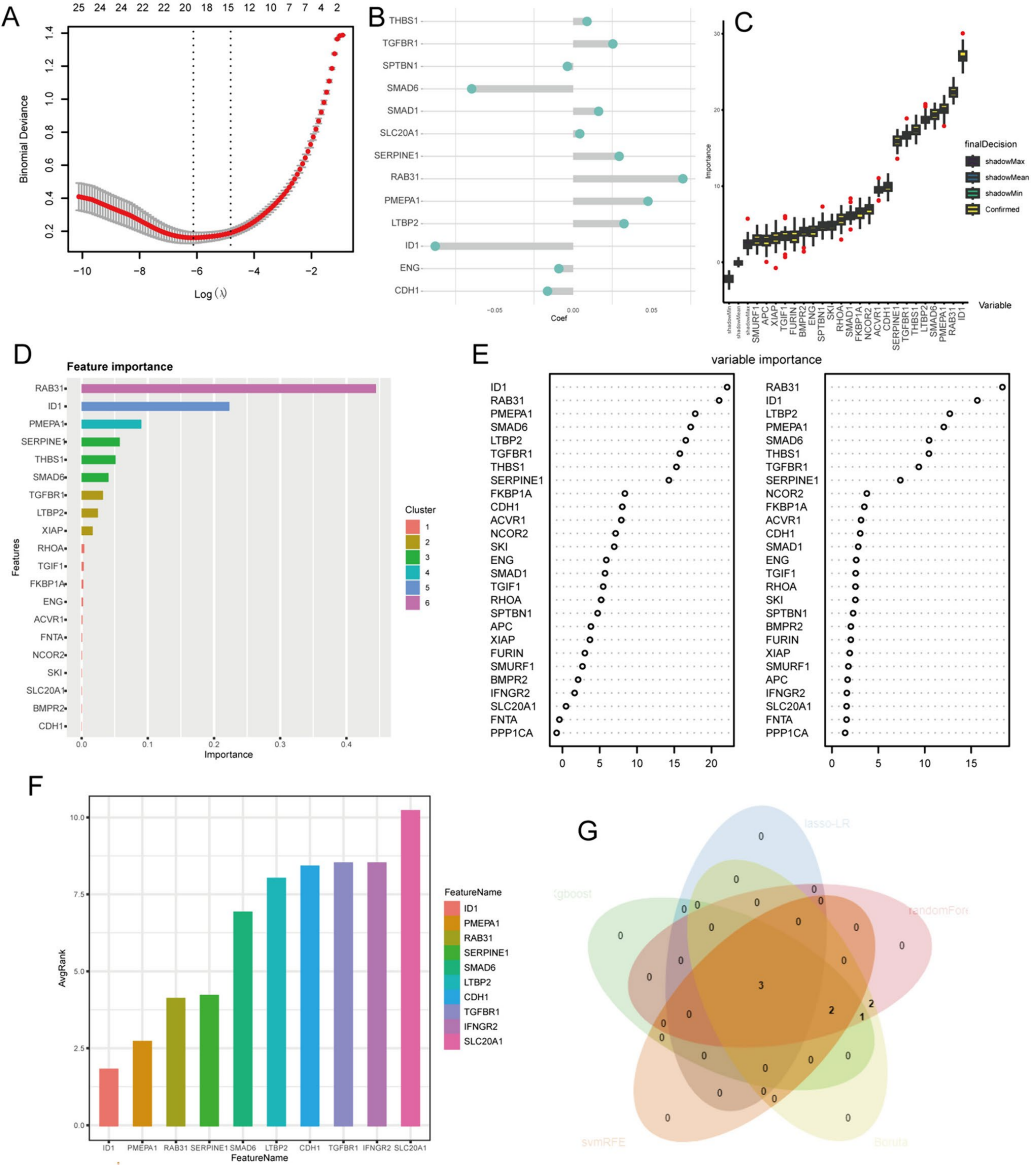
Machine learning identifies SMAD6 as a key gene. **A**–**F** The evaluation of the weighted importance of genes between two clusters by the LASSO-LR (**A**–**B**), Boruta (**C**), XGboost (**D**), random forest (**E**), and svmREF (**F**) algorithm. **G** The intersection of valuable genes from various algorithms

The results above reveal the crucial role of the TGF-β signaling pathway in the development and progression of BCa. In order to precisely identify key genes within the TGF-β signaling pathway, we employed machine learning and prediction on the two populations, identifying the most characteristic gene. Using LASSO-LR, random-forest, Boruta, XGboost, and svmREF algorithms, we filtered 3, 6, 8, 8, and 5 genes, respectively (Fig. 5A–F). By intersecting the 5 algorithms, these three characteristic genes in the intersection corner, are RAB31, ID1, and SMAD6 (Fig. 5G). Finally, we identified SMAD6 as a biomarker of bladder cancer prognosis (Supplementary Fig. 1).

### The landscape of SMAD6 expression in scRNA-seq in BCa

**Fig. 6:**
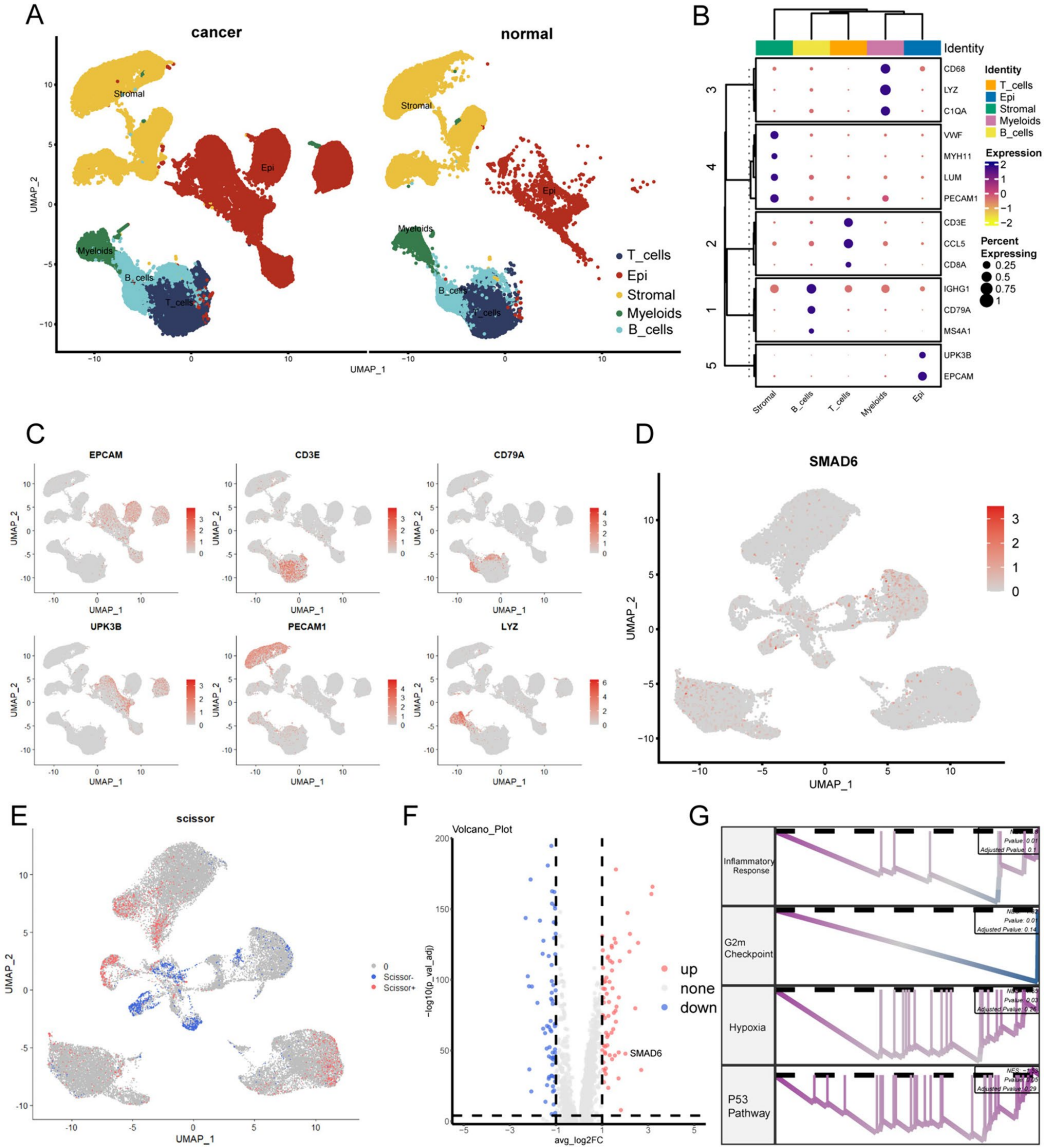
Analysis of SMAD6 based on single-cell sequencing data. **A** UMAP visualization of different cell types in BCa and normal tissue. **B** The dot plot demonstrates classic biomarkers expressed in different cell types. **C** UMAP plot shows the distribution of classic biomarkers. **D** UMAP visualization of the expression of SMAD6 in epithelial cells. **E** The Scissor algorithm identifies the Scissor + / Scissor- cells among epithelial cells. **F** The volcano plot shows differentially expressed genes between Scissor- cells and Scissor + cells. **G** GSEA investigates the biological functions of SMAD6 highly expressed cells (Cells with higher expression of SMAD6 VS cells with lower expression of SMAD6). (UMAP: Uniform Manifold Approximation and Projection)

The single-cell RNA-seq data of 11 samples were reanalyzed from SRP280327. Among these samples, 3 were from normal bladder tissue and eight were from BCa. We annotated five cell types using classical markers: epithelial, stromal, myeloid, B, and T cells (Fig. 6A). After quality control, 99,791 cells were screened, including 40,702 epithelial cells (UPK3B, EPCAM), 24,278 stromal cells (VWF, MYH11, LUM, PECAM1), 4847 myeloid cells (CD68, LYZ, C1QA), 9323 B cells (CD79A, IGHG1, MS4A1) and 20,641 T cells (CD3E, CD8A, CCL5) (Fig. 6B, C) (Becker et al. 2022). we extracted epithelial cells from eight tumor tissues and yielded that SMAD6 expressed in many epithelial cells (Fig. 6D). Then, the Scissor algorithm was employed to identify cells that are related to prognosis. Cells labeled Scissor + were associated with a poor prognosis, while those labeled Scissor- were the opposite (Fig. 6E). After differential analysis between Scissor + cells and Scissor- cells, the volcano plot unraveled that SMAD6 was highly expressed in Scissor- cells (Fig. 6F and Supplementary Table 2).

To further investigate the effect of SMAD6 expression on the biological features of epithelial cells, we conducted GSEA analysis on cells with high and low expression of SMAD6, and the result showed that downregulation of the P53 signaling pathway in cells with higher expression of SMAD6, and their hypoxia and DNA damage was slighter (Fig. 6G).

### Survival and correlation analysis

**Fig. 7:**
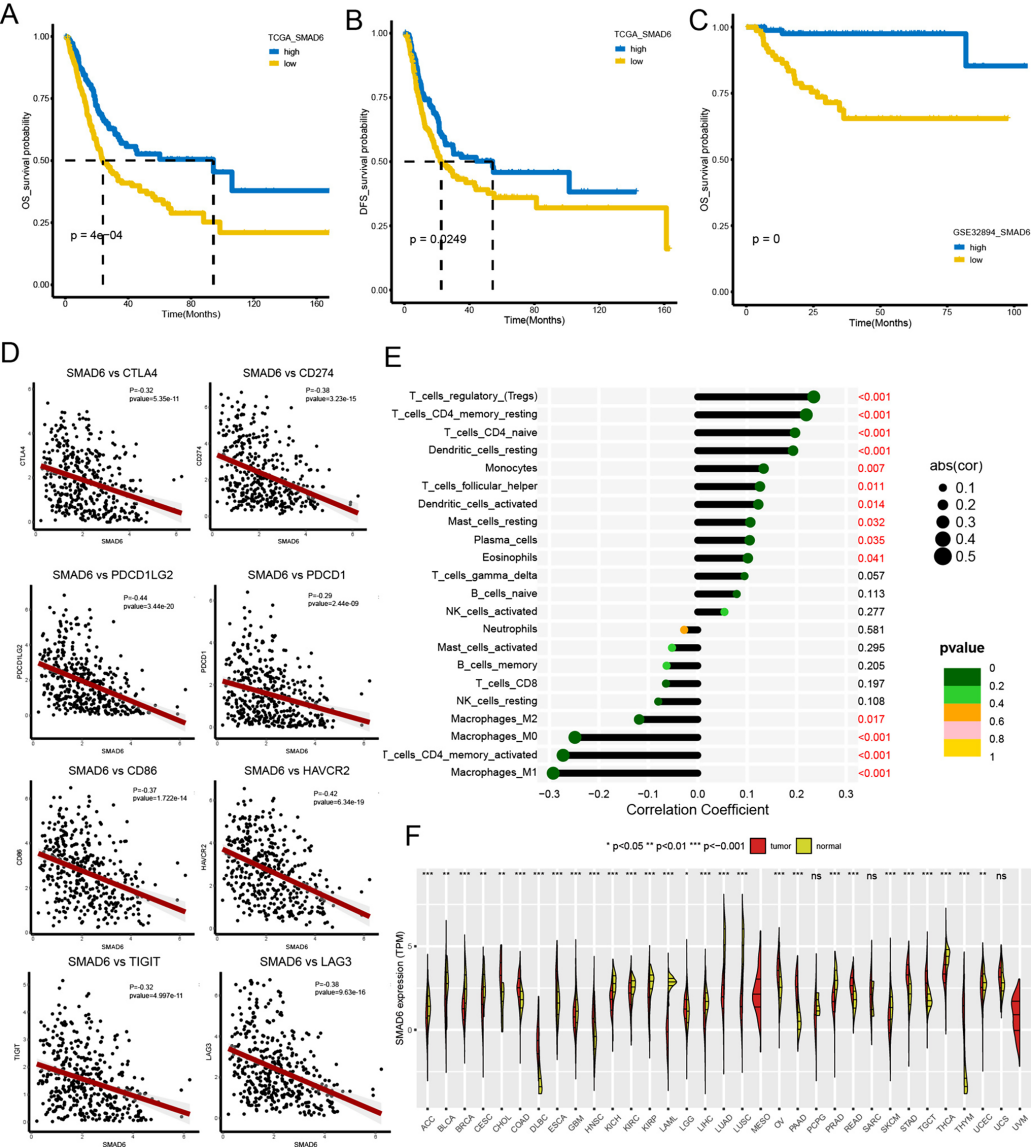
Survival analysis, correlation analysis and pan-cancer analysis of SMAD6. **A-B** The Kaplan–Meier curves demonstrate the prognostic value of SAMD6 in OS (**A**) and DFS (**B**). **C** The survival curve for MIBC patients expressing SMAD6 in GSE32894. **D** The correlation of SMAD6 between immune checkpoint genes. **E** The CIBERSORT algorithm infers the correlation between SMAD6 and immune infiltration. **F** The differential expression of SMAD6 between tumor and normal tissues in pan-cancer. Wilcoxon test: ^*^P < 0.05, ^**^P < 0.01, ^***^P < 0.001, ^****^P < 0.0001

To further reveal the impact of SMAD6 on the survival of patients with BCa, we conducted a survival analysis and yielded that samples with high SMAD6 expression had longer overall survival (OS) and disease-free survival (DFS) in the TCGA cohort among MIBC patients (Fig. 7A, B, p < 0.0001, p = 0.0249). The results demonstrated that high SMAD6 expression represented a better prognosis. Using GSE32894 as validation cohort, the conclusion emerged (Fig. 7C, p < 0.0001).

In correlation analysis, we found a negative correlation between SMAD6 and many immune checkpoint genes, indicating that tumors with low expression of SMAD6 may have an immunosuppressive microenvironment, which could be a reason for their poorer survival outcomes. (Fig. 7D). To further elucidate the relationship between SMAD6 and the immune microenvironment in BCa, we conducted correlation analysis between SMAD6 and immune cells. The results revealed a negative correlation between SMAD6 and tumor-infiltrating macrophages (Fig. 7E). In pan-cancer analysis, SMAD6 exhibits differential expression between various cancers and normal tissues, suggesting its potential role in multiple cancers warrants further investigation (Fig. 7F).

### SMAD6 inhibited the proliferation and invasion ability of BCa cells

**Fig. 8:**
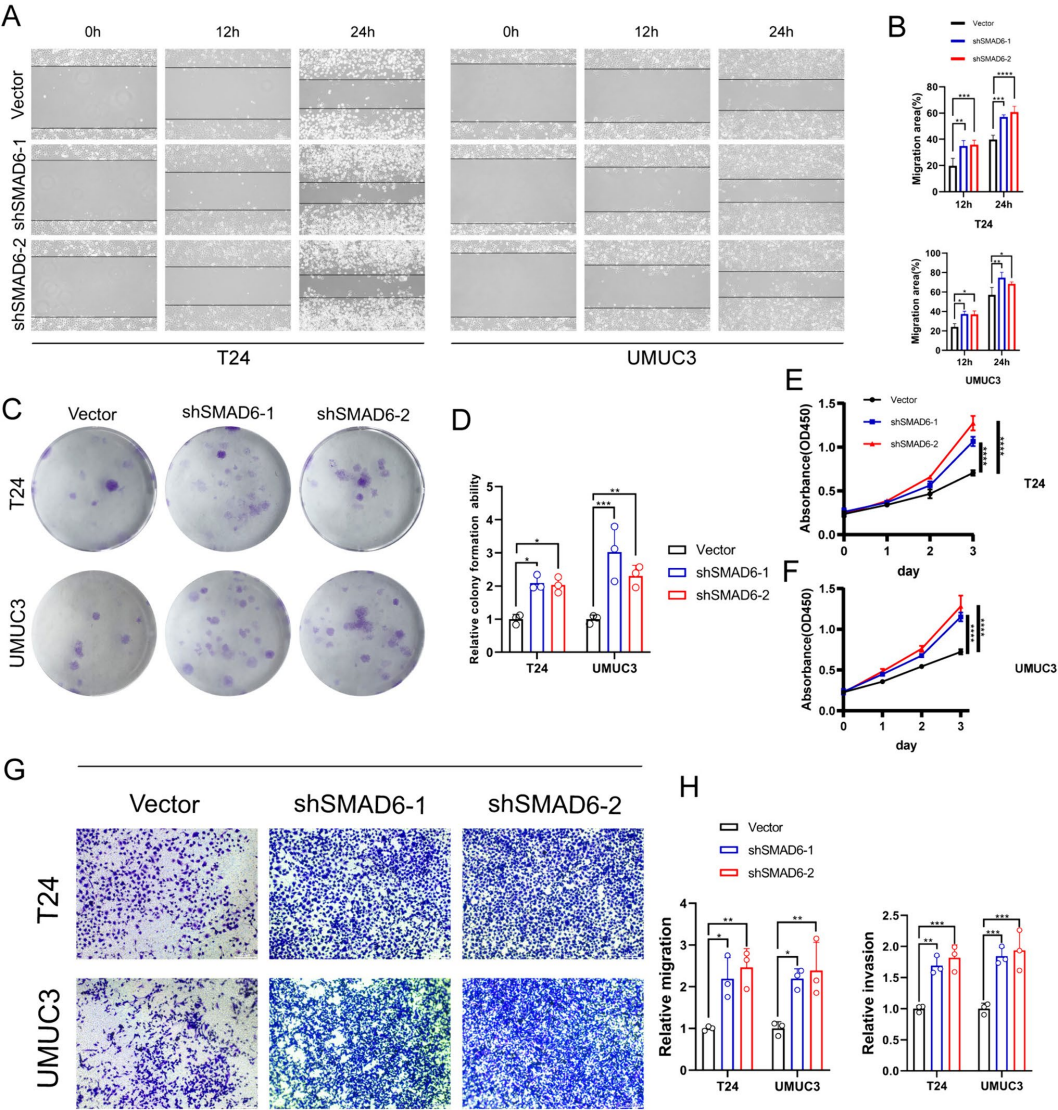
A series of in vitro experiments verifying the function of SMAD6. **A-B** Knock-down of SMAD6 increases the migration ability of BCa cell lines. **C-D** Knock-down of SMAD6 strengthens the colony formation ability of BCa. **E–F** Knock-down of SMAD6 augments the vitality of BCa. **G-H** Knock-down of SMAD6 enhances the migration and invasion ability of BCa. ^*^P < 0.05, ^**^P < 0.01, ^***^P < 0.001, ^****^P < 0.0001

QRT-PCR yielded that the mRNA expression level of SMAD6 in bladder cancer cell lines was significantly lower than in normal bladder cells (Supplementary Fig. 2). To further investigate the role of SMAD6 in BCa, SMAD6-shRNAs were used to transfect T24 and UMUC3 cell lines. Wound healing assays illustrated that the knock-down of SMAD6 obviously increased the migration of BCa (Fig. 8A–B). Colony formation assays detected that the colony numbers were remarkably increased in the shSMAD6-1 and shSMAD6-1 groups (Fig. 8C–D). Besides, CCK-8 assays yielded consistent conclusions as the above experiments (Fig. 8E–F). Finally, the transwell assays displayed that knock-down of SMAD6 dramatically enhanced the migration and invasion ability of BCa (Fig. 8G–H). In conclusion, SMAD6 inhibited the proliferation and invasion ability of BCa in vitro.

## Discussion

Bladder cancer stands as one of the most prevalent malignant tumors affecting the urinary system, posing a substantial burden on global health due to its tendency for recurrence and progression (Antoni et al. 2017). The TGF-β signaling pathway, a prominent pathway in cancer research, has been demonstrated to be associated with drug resistance and the immune microenvironment in bladder cancer (Benjamin and Lyou 2021). In this study, consensus clustering identified two subgroups related to the TGF-β signaling pathway. It was observed that cluster 1 (C1) had a worse prognosis compared to cluster 2 (C2). Furthermore, the upregulation of the TGF-β signaling pathway in C1, as opposed to C2, suggests that the activation of the TGF-β signaling pathway influences the overall survival of bladder cancer patients.

The tumor immune microenvironment is associated with immune checkpoint inhibitor (ICI) therapy, categorizing tumors into immune-inflamed phenotype and immune-desert phenotype based on T-cell abundance and response to ICI treatment (Xiao et al. 2019). Generally, the latter lacks T-cell infiltration and exhibits lower responsiveness to ICI. We quantified the tumor microenvironment of these two subtypes using various algorithms, revealing that C1 has a more complex tumor microenvironment. In comparison to C2, C1’s immune microenvironment appears more “cold,” with less infiltration of T cells, NK cells, and DC cells. TGF-β inhibits CD8 + T cells through direct or indirect regulation, such as suppressing IL-2 expression necessary for CD8 + T cell growth and proliferation, and downregulating perforins, granzymes A/B, and IFNγ to affect CD8 + T cell activity (Monjazeb et al. 2014). Additionally, studies suggest that TGF-β inhibits NK cell activation and cytotoxicity by downregulating NKG2D expression (Fujii et al. 2018). In contrast, macrophage infiltration shows opposite results, as C1 exhibits richer macrophage infiltration compared to C2. Research indicates that TGF-β can suppress the activity of M1 macrophages, the main components exerting anti-tumor immune responses among macrophages (Shapouri-Moghaddam et al. 2018), through the Smad7 and TNF pathways. Beyond immune cells, the TGF-β pathway appears linked to cancer-associated fibroblasts (CAFs) (Wu et al. 2021). C1 shows higher CAF abundance, and recent studies suggest that CAFs promote tumor progression and metastasis by secreting cytokines and chemokines into the extracellular matrix (ECM) and reshaping the ECM. Notably, TGF-β can recruit more fibroblasts into the tumor microenvironment (TME) and induce their transformation into CAFs (Fang et al. 2023). The activated TGF-β signaling pathway in CAFs can also inhibit cell death and play a role in cancer progression (Yoshida 2020).

Bladder cancer molecular subtypes and biological characteristics vary among different subgroups. This study elucidates that the EMT activity is more pronounced in C1, indicating a propensity for progression and metastasis in tumors of C1. Additionally, the tumor microenvironment of C1 exhibits more severe hypoxia, which has been previously associated with tumor progression and drug resistance. We also found that angiogenesis is more active in C1, which is correlated with tumor cell vitality due to its role in nutrient and oxygen supply to tumors (Li et al. 2019). Furthermore, we observed upregulation of the TNFα-NFκB and Kras signaling pathways in the C1 subtype, which have been implicated in the progression and drug resistance of various cancers (Girouard et al. 2020; Timar and Kashofer 2020; Kim et al. 2023). Some research has found that the androgen receptor (AR)-mediated ADAR2/FNTA pathway alters the invasion of BCa cells and their sensitivity to cisplatin by activating the Kras signaling pathway (Chen et al. 2020a). The metabolic landscape of bladder cancer reveals distinct metabolic characteristics between C1 and C2. Methionine metabolism is involved in numerous cellular functions, including redox reactions, folate metabolism, and methylation processes, all of which are implicated in the pathogenesis of cancer (Sanderson et al. 2019). Research has shown that the accumulation of methionine metabolism products may lead to hypermethylation of tumor suppressor genes, thereby increasing the risk of bladder cancer progression (Wojtczyk-Miaskowska and Schlichtholz 2019; Li et al. 2023a). Animal experiments have demonstrated the therapeutic effects of methionine-restricted diets in chemotherapy-resistant colorectal cancer xenograft models (Gao et al. 2019). Additionally, studies have found that methionine-restricted diets can effectively inhibit platinum resistance in bladder cancer patients, thereby enhancing the efficacy of chemotherapy (Yang et al. 2023). The relationship between methionine metabolism and the TGFβ signaling pathway warrants further investigation. At the biochemical level, glutathione metabolism is closely linked to cellular hypoxia, and the higher levels of glutathione metabolism in C1 compared to C2 are consistent with previous findings. Glutathione is one of the most important antioxidants in mammalian cells, protecting cells from damage by reactive oxygen species and lipid peroxidation. The antioxidant system regulated by glutathione is crucial in maintaining the homeostasis of cancer cells (Kennedy et al. 2020). A series of studies have demonstrated the association of glutathione with cancer cell proliferation, differentiation, metastasis, and treatment response. Glutathione metabolism is involved in the metabolic reprogramming of cancer cells during cancer progression. In this process, cancer cells can enhance their tolerance to adverse microenvironments(Estrela et al. 2006). Research has found that glutathione metabolism is associated with a form of regulated cell death induced by excessive lipid peroxidation, known as ferroptosis (Li et al. 2024). Glutathione can promote tumor growth by inhibiting ferroptosis.

Given the differences between the C1 and C2 subtypes of BCa mentioned above, identifying key genes that contribute to these differences may lead us to identify a novel biomarker for BCa. Machine learning classifiers can identify the key factors causing differences based on input expression matrices, with high precision and sensitivity. Combining the results of classification from five machine learning algorithms with survival analysis, we ultimately determined that SMAD6 can serve as a prognostic biomarker for bladder cancer. The SMAD family, as downstream signaling receptors of the TGF-β family, consists of eight members. SMAD6 is a key negative regulator in the classical TGF-β signaling pathway, capable of inhibiting TGF-β receptor activity in the cytoplasm and acting as a transcriptional inhibitory protein in the cell nucleus (Derynck and Zhang 2003). Previous research has suggested that the TGF-β/SMAD pathway induces cell proliferation, angiogenesis, and epithelial-mesenchymal transition (Song and Zhou 2021). Additionally, studies have indicated that inhibiting SMAD signaling can reduce the expression of PD-L1 and PD-L2, implying that the TGF-β/SMAD pathway creates an immune-suppressive microenvironment (MaruYama et al. 2022). However, among the SMAD family members, it remains unclear which one is the key regulatory gene. Through machine learning screening, this study revealed that the TGF-β/SMAD signaling plays an important role in the progression of bladder cancer, with SMAD6 identified as a significant regulator. To elucidate the role of SMAD6 in bladder cancer, single-cell data analysis revealed a correlation between SMAD6 expression and clinical outcomes, predicting patients' overall survival (OS). Simultaneously, GSEA results demonstrated that biological processes such as the P53 pathway and G2M checkpoint are more active in epithelial cells with low SMAD6 expression. The role of the G2M checkpoint is to prevent cells with genomic DNA damage from entering the mitotic phase, indicating that cancer cells with low SMAD6 expression exhibit more severe DNA damage and greater genomic mutation diversity. P53, as a classical tumor-suppressive pathway, plays a crucial role in tumorigenesis when mutated or deactivated. Enrichment analysis results revealed a connection between low SMAD6 expression and p53 mutations. In summary, the overexpression of SMAD6 led to the downregulation of the TGF-β signaling pathway in bladder cancer, thereby inhibiting cancer cell EMT and reducing the progression and metastasis of bladder cancer. Additionally, SMAD6 is associated with the tumor microenvironment, where high SMAD6 expression enhances immune-mediated tumor killing effects. These factors may contribute to SMAD6’s impact on patient survival. The influence of SMAD6 on bladder cancer drug sensitivity may be regulated through alterations in bladder cancer metabolism. Based on metabolism landscape results, SMAD6 likely upregulates methionine metabolism in cancer cells by mediating the TGF-β signaling pathway, leading to cisplatin resistance in cancer cells.

Simultaneously, we also analyzed the role of SMAD6 in various cancers. Survival analysis demonstrated that the role of SMAD6 varies in different cancers. In LUAD, STAD, and KIRC, low expression of SMAD6 implies a worse prognosis, while the opposite is observed in BRCA and COAD. The role of the TGF-β/SMAD signaling pathway has been extensively documented across various cancers (He et al. 2022). SMAD transcriptional regulatory events play complicated roles in the pathogenesis of cancers in the intestinal mucosa, pancreas, and liver (Du et al. 2021). Additionally, the TGF-β/SMAD signaling pathway participates in regulating tumor stromal cells and the immune system (Zhang et al. 2022). Literature has reported that SMAD6 acts as a downstream target of miRNA to regulate the TGF-β signaling pathway, thereby further inhibiting cancer cell proliferation (Bayat et al. 2021).

## Conclusion

In summary, our study reveals two bladder cancer patient subtypes based on the TGF-β pathway. The C1 is associated with a poorer prognosis, a colder immune microenvironment, and higher drug resistance. We identified that SMAD6 plays a crucial regulatory role in the TGF-β/SMAD pathway in bladder cancer, and several biological characteristics of the C1 may be related to the deficiency of SMAD6. Our research emphasizes the importance of the TGF-β/SMAD pathway in the progression of bladder cancer and provides potential targets and strategies for the precise therapy of bladder cancer.

## Data Availability

The data are available from TCGA database (https://portal.gdc.cancer.gov/), the GEO database (http://www.ncbi.nlm.nih.gov/geo/, GSE32984).
